# Comparative analysis of the root transcriptomes of cultivated sweetpotato (*Ipomoea batatas* [L.] Lam) and its wild ancestor (*Ipomoea trifida* [Kunth] G. Don)

**DOI:** 10.1186/s12870-016-0950-x

**Published:** 2017-01-13

**Authors:** Sathish K. Ponniah, Jyothi Thimmapuram, Ketaki Bhide, Venu (Kal) Kalavacharla, Muthusamy Manoharan

**Affiliations:** 1 Department of Agriculture, University of Arkansas at Pine Bluff, Pine Bluff, Arkansas USA; 2 Bioinformatics Core, Purdue University, West Lafayette, Indiana USA; 3 Center for Integrated Biological and Environmental Research (CIBER), Delaware State University, Dover, Delware USA; 4 Molecular Genetics and Epigenomics Laboratory, College of Agriculture & Related Sciences, Delaware State University, Dover, Delware USA

**Keywords:** *Ipomoea batatas*, *Ipomoea trifida*, Storage root, Fibrous root, Transcriptome, Molecular markers

## Abstract

**Background:**

The complex process of formation of storage roots (SRs) from adventitious roots affects sweetpotato yield. Identifying the genes that are uniquely expressed in the SR forming cultivated species, *Ipomoea batatas* (*Ib*), and its immediate ancestral species, *Ipomoea trifida* (*It*), which does not form SRs, may provide insights into the molecular mechanisms underlying SR formation in sweetpotato.

**Results:**

Illumina paired-end sequencing generated ~208 and ~200 million reads for *Ib* and *It*, respectively. Trinity assembly of the reads resulted in 98,317 transcripts for *Ib* and 275,044 for *It,* after post-assembly removal of *trans*-chimeras. From these sequences, we identified 4,865 orthologous genes in both *Ib* and *It*, 60 paralogous genes in *Ib* and 2,286 paralogous genes in *It*. Among paralogous gene sets, transcripts encoding the transcription factor *RKD*, which may have a role in nitrogen regulation and starch formation, and rhamnogalacturonate lyase (*RGL*) family proteins, which produce the precursors of cell wall polysaccharides, were found only in *Ib.* In addition, transcripts encoding a K^+^ efflux antiporter (*KEA5*) and the *ERECTA* protein kinase, which function in phytohormonal regulation and root proliferation, respectively, were also found only in *Ib*. qRT-PCR indicated that starch and sucrose metabolism genes, such as those encoding ADP-glucose pyrophosphorylase and beta-amylase, showed lower expression in *It* than *Ib*, whereas lignin genes such as caffeoyl-CoA O-methyltransferase (*CoMT*) and cinnamyl alcohol dehydrogenase (*CAD*) showed higher expression in *It* than *Ib.* A total of 7,067 and 9,650 unique microsatellite markers, 1,037,396 and 495,931 single nucleotide polymorphisms (SNPs) and 103,439 and 69,194 InDels in *Ib* and *It,* respectively, were also identified from this study.

**Conclusion:**

The detection of genes involved in the biosynthesis of *RGL* family proteins, the transcription factor *RKD*, and genes encoding a K^+^ efflux antiporter (*KEA5*) and the *ERECTA* protein kinase only in *I. batatas* indicate that these genes may have important functions in SR formation in sweetpotato. Potential molecular markers (SNPs, simple sequence repeats and InDels) and sequences identified in this study may represent a valuable resource for sweetpotato gene annotation and may serve as important tools for improving SR formation in sweetpotato through breeding.

**Electronic supplementary material:**

The online version of this article (doi:10.1186/s12870-016-0950-x) contains supplementary material, which is available to authorized users.

## Background

Sweetpotato (*Ipomoea batatas* [L.] Lam.) is a key food crop worldwide, with high levels of vitamin A and other essential nutrients; sweetpotatoes also produce large quantities of biomass suitable for conversion to bioethanol [1]. Sweetpotato storage roots (SRs) function in carbohydrate storage and vegetative propagation [2, 3] and form from adventitious roots. Adventitious roots develop from nodal primordia and cut ends or wounds of stem (slips) at 5–15 days after transplanting. These adventitious roots can then form SRs by a process that involves thickening of the vascular tissue, followed by the accumulation of starch and proteins [4]. Adventitious roots can also form fibrous roots (FRs), which undergo lignification of the stele; in contrast to FRs, SRs do not undergo stele lignification [4–6]. The conversion of adventitious roots to SRs involves the formation of new cambial cells, followed by the development of secondary cambium and thin-walled parenchyma cells. Despite its importance, key factors in SR development remain to be discovered.

Although the molecular mechanism underlying the transition from adventitious roots to SRs in sweetpotato is not yet clear, substantial prior work has implicated the plant hormones cytokinin, auxin, and abscisic acid (ABA) in the formation and thickening of SRs [7–11]. For example, ABA functions in the secondary thickening of vascular cambium during SR formation in sweetpotato [10]. Transcription factors from various families have also been implicated in SR formation. For example, the transcription factor gene *IbMADS1* (*Ipomoea batatas MADS-box 1*) is expressed during the early stages of SR initiation [12]; also, cytokinins and jasmonic acid induce the expression of *IbMADS1*. Noh et al. [11] isolated a cDNA encoding the MADS-box protein *SRD1*, which plays an important role in the formation of SRs by activating the proliferation of cambium and metaxylem cells to induce the initial thickening of SRs. The expression of *SRD1* is regulated by the auxin indole-3-acetic acid. Also, overexpression of the class I knotted1-like homeobox (*KNOX1*) genes, *Ibkn1* and *Ibkn2,* results in increased cytokinin activity in sweetpotato, indicating that *KNOX1* functions in controlling cytokinin levels in SRs [13].

Expression analysis during SR formation also identified a number of candidate genes [14–16]. For example, You et al. [14] identified 22 differentially expressed genes by comparing early SRs and fibrous roots. Several NAC family transcription factor genes are downregulated in SRs, and two NAM-like genes, as well as sporamin genes and genes involved in starch biosynthesis, are upregulated in SRs (compared to FRs) at six weeks after planting [15]. Noh et al. [16] used antisense RNA interference to demonstrate the negative role of an expansin gene (*IbEXP1*) in SR development; *IbEXP1* suppresses the proliferation of metaxylem and cambium cells, and thus inhibits the initial thickening of SRs.

Recent work used microarray and next-generation sequencing technologies to examine the molecular mechanism of SR formation in sweetpotato. Wang et al. [17] used microarray analysis to identify transcription factors involved in SR development, such as DA1-related proteins, SHORT-ROOT, and BEL1-like proteins. Using Illumina sequencing, Tao et al. [18] identified genes that are differentially expressed at different stages of sweetpotato root formation. In particular, they found that a gene encoding sucrose phosphate synthase, which functions in sucrose metabolism, is highly expressed in SRs than in fibrous roots. Firon et al. [2] analyzed the root transcriptomes of sweetpotato SRs and non-storage/fibrous roots and demonstrated that phenylpropanoid pathway genes, such as those encoding coumaroyl CoA-synthase and phenylalanine ammonia lyase, are downregulated during the conversion of FRs to SRs, whereas starch metabolism genes, such as those encoding ADP-glucose pyrophosphorylase and starch synthase, are upregulated in SRs.

The cultivated sweetpotato likely evolved from the wild tetraploid *I. trifida* and diploid *I. trifida/I. tabascana* species [19–22]; these wild relatives do not form SRs. Previous transcriptome analyses investigating SR formation examined only the hexaploid cultivated species [2, 23, 24]. Therefore, comparative transcriptome analysis of the wild and cultivated species of sweetpotato may advance our knowledge on the mechanism underlying SR formation in this important crop. In this study, we performed transcriptome analysis of the roots from cultivated sweetpotato (*Ib; Ipomoea batatas* [L.] Lam) and its non-tuber forming relative (*It; Ipomoea trifida* [Kunth] G. Don) to elucidate possible pathways and candidate genes involved in SR formation.

## Results and discussion

### *De novo* assembly of root transcriptomes using Illumina sequencing

**Fig. 1:**
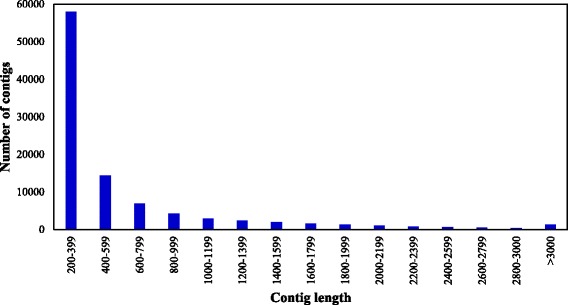
Length distribution of *I. batatas* contigs. Total reads were assembled using Trinity and grouped based on sequence length at 200-bp intervals

**Fig. 2:**
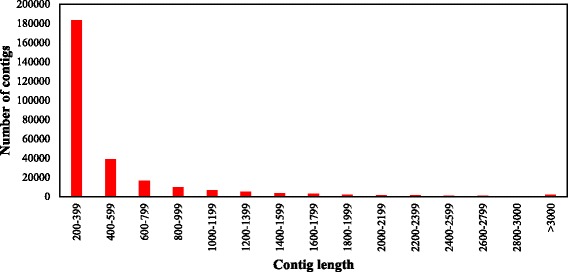
Length distribution of *I. trifida* contigs. Total reads were assembled using Trinity and grouped based on sequence length at 200-bp intervals

High-throughput sequencing of the root transcriptomes of cultivated *Ipomoea batatas* (*Ib*) and its wild ancestor *Ipomoea trifida* (*It*) generated 416 and 400 million reads, respectively. All reads were assembled using Trinity (Version r2012–10–15) with default parameters. The maximum contig length was 14,381 bp for *Ib* and 15,897 bp for *It* (Table 1). The contigs were grouped based on sequence length at an interval of 200 base pairs (bp). The majority of contigs were ranged from 200–399 bp in length (59.0% for *Ib* and 66.7% for *It*), followed by 400–599 bp (14.7% for *Ib* and 14.1% for *It*). In addition, 25.0 and 18.6% of the contigs were 600–3000 bp in length for *Ib* and *It* respectively, and only 1.3% of the contigs in *Ib* and 0.6% of the contigs in *It* were >3000 bp long (Figs. 1 and 2).

**Table 1:** Summary of RNA-seq reads from *Ib* and *It*

Genotype	No. of reads (PE, 2×100)	No. of contigs	N_50_ contig length	Min. length of contigs	Max. length of contigs	Filtered transcripts^a^
*I. batatas* (*Ib*)	208,213,047	240,915	1,791	201	14,381	98,317
*I. trifida* (*It*)	200,191,376	366,513	1,217	201	15,897	275,044

^a^Selected highly covered isoforms using RSEM and post-assembly *trans*-chimera cleanup using BLASTX results against the non-redundant protein database

### Functional annotation by sequence comparison with public databases

To perform functional annotation of the *Ib* and *It* contigs, we used BLASTX [25] with a cutoff e-value of 1.0E-3 to search public databases, such as the non-redundant (nr), Arabidopsis, cassava, and potato databases, and the sweetpotato gene index. This analysis showed that 44.1% of *Ib* transcripts and 63.8% of *It* transcripts matched the non-redundant (nr) database, but only 33.0% of *Ib* and 32.7% of *It* transcripts matched the Arabidopsis database. Similarity searches of the sweetpotato gene index revealed matches of only 25.7% and 8.7% of transcripts for *Ib* and *It*, respectively. The low percentage of *Ib* and *It* transcripts with sequence similarity to sweetpotato gene index may be attributed to the limited number of annotated genes in the database. Searches of the potato and cassava databases also showed the same degree of similarity as found for searches of the Arabidopsis database (Table 2). Overall, 51.7% of the transcripts from *Ib* and 66.4% of the transcripts from *It* showed homology with at least one database. The detailed functional annotation for each species is presented in Additional files 1 and 2.

**Table 2:** BLAST annotation of *Ib* and *It* transcripts

Number of transcripts with matches in:	*Ib*	*It*
Sweetpotato gene index	25,258	23,909
NCBI nr database	43,369	175,495
Arabidopsis database	32,427	89,972
Cassava database	33,006	90,308
Potato database	33,335	87,150
At least one database	50,860	182,692
No matches in any database	47,457	92,352
Information in Trinotate	11,237	25,068

### Comparative analysis of gene sets between *Ib* and *It*

To allow direct comparison of loci between *Ib* and *It*, we used the annotated transcripts to identify orthologous and paralogous genes in *Ib* and *It*. Among the 60 paralogous gene sets with 148 contigs identified in *Ib*, 18 contigs had no match in the Arabidopsis database, whereas the remaining 130 transcripts that matched with the Arabidopsis database were found to be involved in cellular organization, membrane transport, glucose metabolic activity, and carbohydrate metabolism. However, none of the 60 paralogous gene sets identified in *Ib* had matches in the sweetpotato gene index, most likely due to the small number of annotated genes available in this database (Additional file 3). In *It,* we identified 2,286 paralogous gene sets with 9,585 contigs*.* Among 9,585 contigs, 4,890 contigs had no match in the Arabidopsis database; the remaining 4,695 transcripts, which matched with the Arabidopsis database, were involved in transporter activity, stress-related functions, and ribosomal proteins involved in translation. In contrast, only 72 transcripts, which matched with sweetpotato gene index, showed annotated gene functions (Additional file 4). We also identified 5,695 and 6,289 transcripts in *Ib* and *It*, respectively, from 4,865 orthologous gene sets. Examples of orthologous genes include calcium-dependent lipid binding (CaLB) domain and the porcino tubulin-binding cofactor, which are involved in stress and defense responses. CaLB-domain genes are upregulated in drought-stressed sweetpotato [26]. The transcription factors observed among the orthologs include *KNOX*, BEL-1 like homeodomain, and NAC domain-containing proteins that are involved in DNA binding and transcription activities. In sweetpotato, *KNOX*1 is involved in secondary thickening of the SRs through enhanced cytokinin activity [13] and BEL-1 like homeodomain involved in SR development [17]. The NAC domain-containing proteins are upregulated in sweetpotato SRs compared to fibrous roots [15]. Overall, the orthologous genes and transcription factors in both *Ib* and *It* are involved in activities such as nucleic acid binding, defense responses, cell division and differentiation, transport, root gravitropism, hormonal control, and glucose metabolism (Additional file 5).

Among paralogous gene sets, genes encoding the rhamnogalacturonate lyase (*RGL*) family proteins, K^+^ efflux antiporter (*KEA5*), and *ERECTA* protein kinases were found only in *Ib. RGL* proteins comprise the major components of pectin polysaccharides in the cell wall [27]. *RGL* proteins also serve as signaling molecules for pectin polysaccharides [28], and are involved in cell wall modifications such as cell wall expansion, porosity, and textural changes during fruit ripening [29–32]. In Arabidopsis, the expression of *RGL* genes, which are involved in lateral root and root hair formation, is altered in response to the inhibition of primary root growth [33]. Also, in potato (*Solanum tuberosum*), overexpression of *RGL* genes leads to distinct morphological changes in the cortex and periderm [34]. The expression of *RGL* genes (with FPKM (Fragments per Kilobase of Exon per Million Fragments Mapped) value of 181.1) only in *Ib* suggests that pectin polysaccharides may play a role in cell expansion leading to the accumulation of storage proteins in developing SRs and that *RGL* proteins may have important roles in the secondary thickening of cell walls.

K^+^ (*KEA5*) transporter genes (with FPKM value of 14.9) are another group of genes that are expressed specifically in *Ib*. These transporters are involved in stomatal activity, leaf movements, ion transport, and the regulation of phytohormones such as auxin, ethylene, and jasmonic acid [35, 36]. K^+^ transporters also play an important role in cell expansion associated with turgor pressure in Arabidopsis [37, 38]. The expression of *KEA5* transporter genes in *Ib* suggests that they may play a role in SR formation through the regulation of phytohormones such as auxin. In plant roots, auxin regulates lateral root development and gravitropism [39]. In sweetpotato, auxin regulates the expression of the transcription factor *SRD1,* which is involved in SR formation [11]. Therefore, the coordinated regulation of transporter genes such as *KEA5* and transcription factors such as *SRD1* in auxin regulation may be a possibility during SR formation in sweetpotato. In the present study, in addition to *RGL* family genes and *KEA5*, *ERECTA* protein kinase (with FPKM value of 2.2) was also identified in the paralogous genes of *Ib* but not in *It. ERECTA* protein kinase is a leucine-rich repeat receptor-like kinase (LRR-RLK) involved in the proliferation of organelles. *ERECTA* protein kinases regulate organ shape and inflorescence development in Arabidopsis [40, 41]. Interestingly, receptor-like kinases are involved in lateral root development in Arabidopsis [42] and cell wall-bound kinases are associated with pectin binding in Arabidopsis [43]. *ERECTA*, along with *RGL* family proteins, might represent an important link between the regulation of cell wall structure and SR development in sweetpotato. Among the transcription factors in paralogous genes, *RKD* (RWP-RK domain) belongs to the RWP-RK domain-containing proteins, which are involved in DNA binding and regulation of transcription activity, were expressed in *Ib* but not in *It*. The RWP-RK protein domain is required for embryonic pattern formation [44] and plays a key role in regulating responses to nitrogen availability [45]. Recent work reported that *NIT2*, a member of the RWP-RK family, influences starch and lipid storage in *Chlamydomonas* [46]. In *Arabidopsis thaliana*, *NLP7*, another member of the RWP-RK family, is an early regulator of cellular response to nitrogen assimilation [47]. The unique expression of RWP-RK proteins (with FPKM value of 18.4) only in *Ib* indicate that these proteins may have a role in nitrogen regulation and starch formation during the development of sweetpotato SRs.

### Expression of genes involved in SR formation

**Fig. 3:**
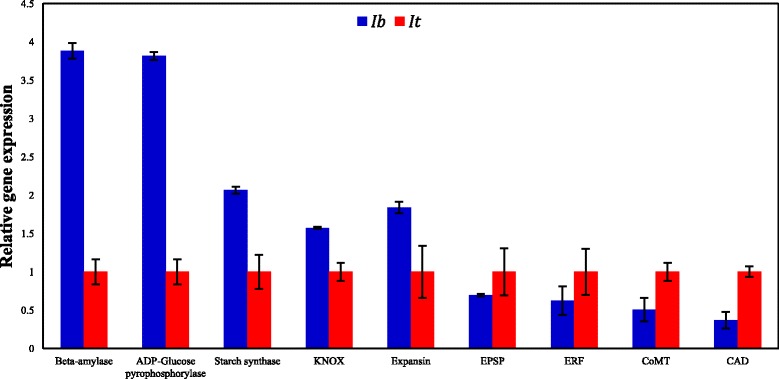
Validation of relative expression levels of selected genes (qRT-PCR). Expression levels were compared between *I. batatas* (*Ib*; indicated in blue) and *I. trifida* (*It*; indicated in red). Quantitative RT-PCR was performed with three biological replicates and two technical replicates for both *Ib* and *It*. The sweetpotato β-*tubulin* gene was used as an endogenous control and the gene expression levels were determined using the ΔΔCt method. Genes are shown with respective TAIR locus IDs: β-amylase (AT4G15210); ADP-glucose pyrophosphorylase (AT4G39210); Starch synthase (AT4G18240); *KNOX*: class I Knotted-like homeobox (AT4G08150); Expansin (AT2G39700); *EPSP*: 5- enolpyruvylshikimate-3-phosphate synthase (AT2G45300); *ERF*: Ethylene-responsive transcription factor (AT1G50640); *CoMT*: caffeoyl-CoA O-methyltransferase (AT4G34050); *CAD*: cinnamyl alcohol dehydrogenase (AT1G72680)

We compared the expression of genes in *Ib* versus *It* based on their respective FPKM values (Table 3). Although two species were compared to estimate up— or down-regulation of gene expression, a clear limitation in our study is the lack of biological replications. Therefore, conclusions reached in this study, may change in future experiments with biological replications. While hundreds or thousands of mapped reads of differentially expressed genes are likely to be reliable, careful reading is required for the contigs/transcripts with few mapped hits. Some evidence for this differential gene expression is supported by the qRT-PCR results (Fig. 3). In the present study, the class-I knotted1-like homeobox gene *KNOX1* was highly expressed in *Ib* (136.9) compared to *It* (50.3). Similarly, Firon et al. [2] observed the increased expression of *KNOX1* in developing sweetpotato SRs versus FRs. *KNOX1* is also associated with higher cytokinin levels, in addition to secondary thickening, in sweetpotato SRs [13, 14]. The cell wall loosening protein expansin was highly represented in *Ib* (70.5) compared to *It* (10.9). Firon et al. [2] demonstrated the involvement of this expansin gene in the initiating SRs of sweetpotato. Also, the gene encoding the sporamin was highly expressed in *Ib* (56,838.3) than in *It* (15.2) (Table 3). Sporamin, a key storage protein in sweetpotato SRs [15], forms in the endoplasmic reticulum, and the mature sporamin moves to the vacuoles of SRs [48]. In sweetpotato, sporamin was accounted for over 80% of the total protein in the SRs [49] and highly expressed in SRs than in FRs [2]. The high expression of sporamin in *Ib* confirmed its role in the synthesis of storage proteins. In the present study, starch and sucrose metabolic genes, such as β-amylase (381.7), glucose-1-phosphate adenylyltransferase (1745.2), phosphoglucomutase (170.2), starch synthase (110.0), ADP-glucose pyrophosphorylase (1752.6), and alpha 1–4 glucan phosphorylase (1305.1) were highly expressed in *Ib*. Similar to our study, the high expression of the gene encoding β-amylase is observed in the initiating SRs compared to fibrous roots in sweetpotato [2]. Also, the increased expression of ADP-glucose pyrophosphorylase is associated with starch accumulation in sweetpotato SRs [18]. Clearly, the high expression of both β-amylase, the second most abundant storage protein in sweetpotato after sporamin [48], and ADP-glucose pyrophosphorylase is positively correlated to their role in starch biosynthesis in sweetpotato [50]. In the present study, we observed higher expression of the gene encoding phosphoglucomutase in *Ib* than in *It*, indicating that the enhanced activity of this enzyme may provide abundant substrate for ADP-glucose pyrophosphorylase, the first step in starch biosynthesis pathway. Indeed, enhanced expression of phosphoglucomutase was previously observed in the SRs of sweetpotato [2]. Another highly expressed gene in *Ib* encodes alpha 1–4 glucan phosphorylase, an enzyme involved in starch phosphorylation in Arabidopsis [51]. The phosphorylation of starch promotes the accumulation of starch granules in the SRs of sweetpotato [18]. The high expression of genes encoding phosphoglucomutase and alpha 1–4 glucan phosphorylase in *Ib* reflects the high activity of starch metabolic genes in SRs.

**Table 3:** Comparison of annotated genes involved in storage root formation between *Ib* and *It* using FPKM values

Annotation	*Ib*	*It*	Ratio
Sporamin	56,838.3	15.2	3739.4
Expansin	70.5	10.9	6.5
Glucose-1-phosphate adenylyltransferase	1745.2	178.7	9.8
Alpha-1,4 glucan phosphorylase	1305.1	0.5	2610.2
Beta-amylase	381.7	28.6	13.3
Phosphoglucomutase	170.2	76.7	2.2
Class-I knotted1-like homeobox protein *Ib*KN2 (*KNOX*)	136.9	50.3	2.7
ADP-glucose pyrophosphorylase	1752.6	178.7	9.8
Starch synthase	110.0	15.9	6.9

### Expression of genes involved in non-storage/fibrous root formation

We compared the expression of genes in *It* versus *Ib* based on their respective FPKM values (Table 4). The results indicate that genes encoding cysteine protease (1709.6), cysteine proteinase (744.9), ethylene responsive transcription factor (*ERF*) (211.8), osmotin-like proteins (787.7), and peroxidase (689.3) were highly expressed in *It*. Cysteine protease is involved in the degradation of sporamin in sprouting sweetpotato SRs [52]. In addition, the cysteine protease gene is downregulated in sweetpotato SRs [2]. In our study, a cysteine protease gene was highly expressed in *It*, indicating that this enzyme may prevent the accumulation of sporamin in the roots and helps them remain as fibrous roots. Cysteine proteinases also exhibit protein degradation activity [53]. The cysteine proteinase gene is upregulated in FRs compared to the SRs in sweetpotato [2]. We observed similar high expression of the cysteine proteinase gene in *It*, indicating that this enzyme may be involved in proteolytic activity and promotes the development of FRs. Genes encoding osmotin-like protein were highly expressed in *It* compared to *Ib* (Table 4). The osmotin-like proteins are plant defense proteins [54] that regulate the production of jasmonic acid and ethylene in Arabidopsis [55]. In sweetpotato, the osmotic-like stress response genes are downregulated in the SRs compared to FRs [2]. The ethylene responsive transcription factor (*ERF*) was highly expressed in *It* than *Ib*, indicating the activity of this stress-responsive gene in the fibrous roots. Likewise, genes encoding peroxidase showed decreased expression in *Ib* versus *It*, indicating that peroxidase genes may be involved in phenylpropanoid and lignin (polymerization) biosynthesis pathways [2]. Another stress-response gene, which encodes succinate dehydrogenase, was minimally increased in *It* versus *Ib*; succinate dehydrogenase functions in the production of reactive-oxygen species during stress-related activities [56]. Overall, the higher expression of proteolytic enzymes such as cysteine protease and cysteine proteinase in *It* indicates that these genes may prevent the accumulation of storage proteins in the developing FRs. Moreover, the higher expression of stress-response genes such as those encoding peroxidases promotes the expression of phenylpropanoid pathway genes and the accumulation of lignin in FRs in sweetpotato [2].

**Table 4:** Comparison of annotated genes involved in fibrous root formation between *Ib* and *It* using FPKM values

Annotation	*Ib*	*It*	Ratio
Cysteine protease	44.9	1709.6	38.1
Cysteine proteinase	145.4	744.9	5.1
Osmotin-like protein	20.4	787.7	38.6
Succinate dehydrogenase	41.9	49.0	1.2
Caffeoyl-CoA O-methyltransferase (*CoMT*)	410.7	975.2	2.4
Phenylalanine ammonia-lyase (*PAL*)	87.3	443.0	5.1
Peroxidase	105.5	689.3	6.5
4-coumarate--CoA ligase (4-CL)	30.3	198.7	6.6
Cinnamyl alcoholdehydrogenase (*CAD*)	67.7	762.9	11.3
5-enolpyruvylshikimate-3-phosphate synthase (*EPSP*)	29.1	144.3	5.0
Ethylene responsive transcription factor (*ERF*)	77.9	211.8	2.7

In addition, lignification helps roots remain as non-storage/fibrous roots and prevents the conversion of fibrous roots to SRs. In the current study, we observed higher expression of genes encoding phenylpropanoid and lignin biosynthetic enzymes, such as 5-enolpyruvylshikimate-3 phosphate (*EPSP*) synthase (144.3), phenylalanine ammonia-lyase (*PAL*) (443.0), 4-coumarate-CoA ligase (4-CL) (198.7), cinnamyl alcohol dehydrogenase (*CAD*) (762.9), peroxidase (689.3), and caffeoyl-CoA O-methyltransferase (*CoMT*) (975.2) in *It* (Table 4). The enzyme EPSP synthase produces an important precursor in the shikimate pathway, promotes the synthesis of lignin and phenylalanine [57]. Consistent with our observations, other studies showed that in sweetpotato, genes encoding cinnamyl alcohol dehydrogenase, coumaroyl-CoA synthase, and caffeoyl-CoA O-methyltransferase are downregulated during SR initiation [2]. Moreover, lignin biosynthesis genes are upregulated in the early stages of fibrous root formation in sweetpotato [17]. The increased expression of the phenylpropanoid and lignin genes in *It* provides evidence for the accumulation of lignins during FR development.

### Validation of gene expression through quantitative reverse-transcription PCR (qRT-PCR)

We profiled different genes, based on FPKM values, for expression in *Ib* and *It* with the RNA samples used for Illumina sequencing. Genes involved in starch and sucrose metabolism, and lignin biosynthesis, such as those encoding ADP-glucose pyrophosphorylase, β-amylase, starch synthase, cinnamyl alcohol dehydrogenase (*CAD*), and caffeoyl-CoA O-methyltransferase (*CoMT*) were selected for qRT-PCR. The genes and their respective primers are presented in Additional file 6. The qRT-PCR results indicated the starch metabolic genes such as ADP-glucose pyrophosphorylase, beta-amylase, and starch synthase were highly expressed in *Ib* than in *It* (Fig. 3). The high expression of starch metabolism genes in *Ib* reflects the movement of storage proteins during SR development. A similar finding for starch metabolism genes was previously reported in sweetpotato [2]. The qRT-PCR results showed that the gene encoding expansin was more highly expressed in *Ib* than in *It*. By contrast, Noh et al. [16] found that the expression of the expansin gene is inhibited during SR formation in sweetpotato. However, Firon et al. [2] demonstrated that the expansin gene is involved in SR formation in sweetpotato. In the present study, the meristematic regulatory gene *KNOX*1 was highly expressed in *Ib*, which was previously demonstrated in the development of sweetpotato SRs [13]. The qRT-PCR results showed that the gene ethylene response factor (*ERF*) was expressed at higher levels in *It* than in *Ib*. By contrast, Firon et al. [2] showed high expression of *ERF* in sweetpotato SRs compared to FRs of the cultivar Georgia Jet. The shikimate pathway gene encoding *EPSP* synthase was highly expressed in *It*. This enzyme forms an important precursor in the shikimate pathway and promotes the biosynthesis of lignin [57]*.* The qRT-PCR results showed that the lignin biosynthetic genes *CAD* and *CoMT* were highly expressed in *It* compared to *Ib*. In summary, the high expression of starch and sucrose metabolism genes in *Ib* promotes SR formation, whereas the high expression of lignin biosynthetic genes in *It* promotes the development of FRs/non-storage roots.

### Functional classification of transcripts using Gene Ontology (GO) and pathway analysis

**Fig. 4:**
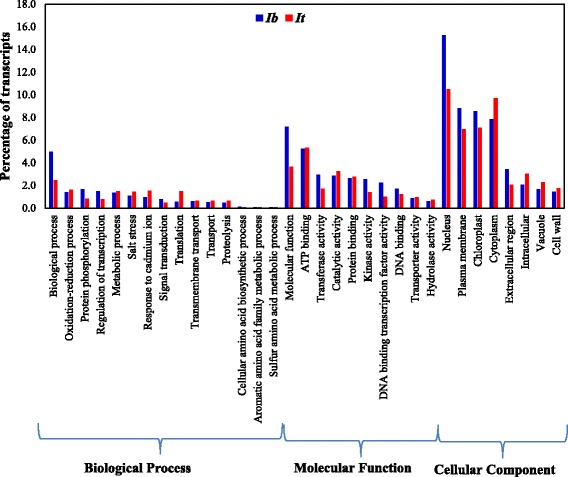
Functional classification of transcripts using Gene Ontology. *I. batatas* (*Ib*) transcripts are indicated in *blue*, and *I. trifida* (*It*) transcripts are indicated in *red*. The non-redundant transcripts were subjected to functional classification using GOSlim

The *Ib* and *It* annotated transcripts were grouped into three biological functions such as biological process, molecular function, and cellular component using the GO Slim database [58]. The majority of GO annotations (47.7% in *Ib* and 46.6% in *It*) were grouped into the biological process category, followed by molecular function (27.8% in *Ib* and 25.6% in *It*). In addition, 24.5% of *Ib* and 27.8% of *It* transcripts were grouped in the cellular component category (Fig. 4; Additional files 1 and 2). Most transcripts in the biological process category are involved in oxidation-reduction processes, protein phosphorylation, regulation of transcription, metabolic processes, salt stress, and signal transduction and translation. The transcripts in the molecular function category are involved in ATP binding, protein binding, DNA binding, phosphorus transferase, and catalytic activity. The transcripts grouped within the cellular component category were based on their predicted sub-cellular locations in the nucleus, plasma membrane, chloroplast, cytoplasm, extracellular space, and vacuole. Similar groupings of annotated genes have been reported in previous sweetpotato root transcriptome studies [2, 24].

**Fig. 5:**
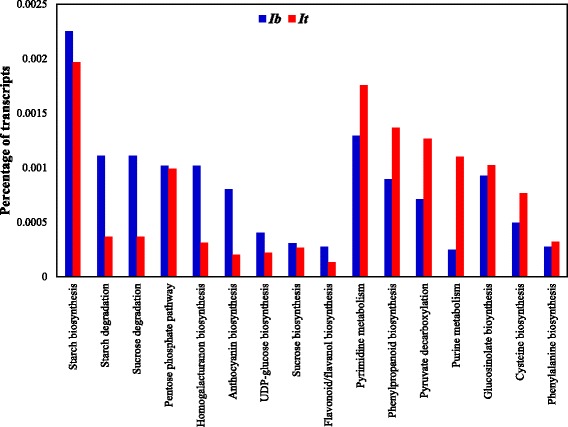
Functional classification of transcripts using DAVID. *I. batatas* (*Ib*) transcripts are indicated in *blue*, and *I. trifida* (*It*) transcripts are indicated in *red*. The functional grouping was based on KEGG pathway names associated with transcripts from DAVID analysis

The annotated transcripts between *Ib* and *It* were assigned to various biological pathways using DAVID [59, 60]. A total of 32,427 (33.0%) and 89,972 (32.7%) transcripts were assigned to 365 and 371 pathways in *Ib* and *It* respectively. Further, we also examined the pathways that are differentially enriched in *Ib* and *It.* The enriched transcripts in *Ib* and *It* (percentage of expressed transcripts) showed 16 major pathways that are involved in storage/fibrous roots (Fig. 5). The list of transcripts and KEGG-enriched pathways from DAVID are presented in Additional files 7 and 8. In *Ib*, we observed a higher percentage of differentially expressed transcripts in the starch (77 transcripts, 0.002% of the expressed transcripts) and sucrose biosynthetic pathways (10 transcripts, 0.001%) compared to *It* (177 transcripts, 0.001% in starch, and 24 transcripts, 0.0002% in sucrose biosynthetic pathways). The increased expression of sucrose and starch metabolic genes indicated the synthesis of sucrose and starch in the storage roots [2]. We also detected a large percentage of transcripts in *Ib* (13 transcripts, 0.0004%) than in *It* (20 transcripts, 0.0002%) for UDP-glucose biosynthesis, which is involved in the synthesis of UDP glucose. The increased expression of transcripts for UDP-glucose biosynthesis in *Ib* indicates the increased activity of UDP sugars in the storage roots. In *Ib*, enrichment of transcripts involved in the homogalacturonan pathway (33 transcripts, 0.001%) was observed in comparison with *It* (28 transcripts, 0.0003%), which indicates the accumulation of pectins in the storage roots. The pectin in the primary cell walls forms from homogalacturonans, rhamnogalacturonan-I, and rhamnogalacturonan-II [28], and strengthens the cell wall of the developing SRs.

The percentage of transcripts of secondary metabolic pathways such as the anthocyanin (26 transcripts, 0.0008%) and flavonoid biosynthetic pathways (9 transcripts, 0.0003%) were more represented in *Ib* than *It* (18 transcripts, 0.0002%, and 12 transcripts, 0.0001%). This is in contrast to the results of Firon et al. [2] who reported higher expression of anthocyanin and flavonoids in FRs than in SRs of sweetpotato. The more representation of anthocyanin and flavonoids in our study could be due to the use of orange-fleshed Beauregard variety. Earlier transcriptome studies showed that genes related to production of anthocyanin and flavonoid compounds were highly expressed in purple and yellow-colored sweetpotato SRs [61].

The transcripts representing the phenylpropanoid pathway (123 transcripts, 0.001%) and phenylalanine biosynthesis (29 transcripts, 0.0003%) were more represented in *It* than *Ib* (29 transcripts in *It*, 0.0008%, and 9 transcripts in *Ib*, 0.0002%) indicating the synthesis of lignin in the fibrous/non-storage roots. Similar results of higher phenylalanine biosynthesis were observed in the FRs of sweetpotato [2]. Transcripts of the pyruvate decarboxylation pathway were also more represented in *It* (114 transcripts, 0.001%) than *Ib* (23 transcripts, 0.0007%). The pyruvate decarboxylation pathway participates in carbohydrate metabolism and down-regulates the synthesis of glucose. In sweetpotato, Firon et al. [2] demonstrated the downregulation of pyruvate decarboxylase in the SRs in comparison with the FRs. We also observed more representation of transcripts related to biosynthesis of glucosinolate (92 transcripts, 0.001%) and cysteine (69 transcripts, 0.0007%), which are involved in stress and defense responses, in *It* compared to *Ib* (30 transcripts, 0.0009%, and 16 transcripts, 0.0004%). In Arabidopsis, the expression of glucosinolate occurs in the antifungal defense response [62]. In sweetpotato, Firon et al. [2] showed the high representation of cysteine biosynthesis genes, consistent with the results of our study, in the non-storage/fibrous roots. In addition, transcripts of purine and pyrimidine metabolism, which are involved in the synthesis of nucleic acids and energy carriers in the cell [63], were more represented in *It* (99 transcripts, 0.001%, and 158 transcripts, 0.002%) than *Ib* (8 transcripts, 0.0002%, and 42 transcripts, 0.001%).

### Identification of Simple Sequence Repeat (SSR) markers

We predicted potential microsatellite markers based on the assembled transcripts generated from *Ib* and *It* using SSRfinder [64] and MISA (Version 1.0) [65]. We mined for new microsatellite markers in *Ib* and *It* using 98,317 and 275,044 transcripts, respectively. A total of 20,065 and 26,158 microsatellite markers were identified in *Ib* and *It*, respectively (Table 5). The list of identified SSRs, along with the associated forward and reverse primers are presented in Additional file 9 (*Ib*) and Additional file 10 (*It*). Overall, the results indicate that di-nucleotide repeats (GA/TA) were most abundant in both *Ib* and *It*. The percentage difference in the number of SSRs identified with di-, tri-, and tetra-nucleotide repeats was higher in *It* than in *Ib* (30.8%, 43.2%, and 52.5% higher, respectively). Functional genetic markers such as SSRs are useful in understanding the genetic variation in plants [66, 67].

**Table 5:** Number of SSRs predicted in *Ib* and *It*

Number of SSRs	*Ib*	*It*
SSRs predicted by MISA	20,065	26,158
SSRs with unique sets of designed primers	7,067	9,650

### Prediction of Single Nucleotide Polymorphisms (SNPs) and insertions and deletions (InDels) between *Ib* and *It*

The reads of *Ib* were mapped to the transcripts of *It* to identify the unique SNPs for *Ib* and vice versa for *It*. A total of 1,037,396 (*Ib*) and 495,931 (*It*) unique SNPs were identified for each species (Table 6). Within orthologous genes, 254,120 and 89,809 SNPs were identified in *Ib* and *It*, respectively. Also, 5,669 (*Ib*) and 883 (*It*) SNPs were identified within paralogous genes. A list of SNPs is provided in Additional file 11 for *Ib* and Additional file 12 for *It*. SNPs and InDels, the most abundant genetic variations in the genome, are often exploited for high-throughput genotyping and marker-assisted selection in plants [68]. Recently, Hirakawa et al. [69] reported a list of SNPs in the *It* genome. In the current study, we identified 103,439 and 69,194 InDels between *Ib* and *It*. In addition, 18,655 and 11,559 InDels were identified within orthologs between *Ib* and *It*, respectively. Within paralogs, 197 and 32 InDels were identified in *Ib* and *It*, respectively (Table 6). A list of InDels is provided in Additional file 13 for *Ib* and Additional file 14 for *It*.

**Table 6:** Number of SNPs and InDels predicted between *Ib* and *It*

Number of SNPs and InDels	*Ib*	*It*
Total SNPs	1,037,396	495,931
SNPs within orthologs	254,120	89,809
SNPs within paralogs	5,669	883
Total InDels	103,439	69,194
InDels within orthologs	18,655	11,559
InDels within paralogs	197	32

## Conclusion

We compared the root transcriptomes of SR forming cultivated *I. batatas* with its non-tuber-forming wild ancestor, *I. trifida*. Among paralogous gene sets, genes encoding *RGL* proteins were identified only in *Ib*; we speculate that *RGL* family proteins may play a role in SR formation in sweetpotato. In addition to the expression of the transcription factor RWP-RK domain-containing protein in *Ib*, other genes that are expressed in *Ib*, such as those encoding K^+^ transporters and *ERECTA* protein kinases, may also play a role in SR formation. qRT-PCR indicated that starch and sucrose metabolism genes such as those encoding ADP-glucose pyrophosphorylase, beta-amylase, and starch synthase, showed enhanced expression in *Ib*. By contrast, lignin biosynthetic genes, such as *CAD* and *CoMT*, were highly expressed in *It* than *Ib*. The root transcriptome data obtained in this study may serve as a resource for the development of molecular markers in sweetpotato and may facilitate annotation of the sweetpotato genome.

## Methods

### Plant materials and growth conditions

Five different *I. batatas* (L.) Lam cv. Beauregard (*Ib*) plants were grown in pots under greenhouse conditions at the University of Arkansas at Pine Bluff (UAPB), Pine Bluff, AR. Seeds from the non-tuber-forming ancestor *I. trifida* (Kunth) G. Don (*It*) (PI 540715) were obtained from the USDA-GRIN germplasm center (Plant Genetic Resources Conservation Unit, Griffin, GA). *It* seeds were scarified using 50% sulfuric acid and rinsed for five minutes in sterile water. The scarified seeds were germinated on sterile paper and the plants were transplanted in pots and grown under greenhouse conditions. Total roots were collected from 100-day-old plants of *Ib* and *It*. The FRs and SRs from five *Ib* plants were cleaned, pooled and frozen in liquid nitrogen. Similarly, the total roots of five *It* plants were cleaned, pooled and frozen for RNA isolation.

### RNA extraction

Total RNA was extracted from roots of *Ib* (designated as *Sp1*) and *It* (designated as *Sp2*) using Qiagen RNeasy kit (Qiagen, Valencia, CA). RNA samples were subjected to DNase I (1 U/μl) treatment for 15 min., followed by heat inactivation at 65 °C for 10 min. The extracted RNA was quantified using NanoDrop 2000c (Thermo Fisher Scientific, Wilmington, DE), and the quality was checked by RNA gel electrophoresis (Additional file 15).

### cDNA library construction and transcriptome sequencing

The cDNA library construction and sequencing of RNA from *Ib* and *It* (without biological controls) were carried out at the UC Davis Genome Center, San Diego, CA. The cDNA libraries were constructed following the manufacturer’s protocol (Illumina, Inc., San Diego, CA). Transcriptome sequencing was carried out using the HiSeq 2500 platform (Illumina, Inc., San Diego, CA).

### Processing of RNA-seq reads

The quality of reads was assessed using FastQC (Version 0.10.0). All reads, after removing the adapter, were used for assembly without quality filtering, since at least 90% of reads passed the minimum quality score of Q30. Reads from *Ib* (*Sp1*) and *It* (*Sp2*) were assembled separately with Trinity (Version r2012– 10– 15) using default parameters [70]. Trinity assembly of *Ib* reads resulted in 240,915 contigs while *It* reads resulted in 366,513 contigs. The reads were mapped back to the assembled transcripts and isoform percentage was calculated using Trinity-RNA-seq by Expectation Maximization (RSEM) [70]. Redundant contigs were filtered from *Ib* and *It* using an optimized assembly method [71] in two steps: i) selecting a highly covered isoform for each unique component-subcomponent from Trinity output based on the RSEM results and ii) post-assembly trans-chimera cleanup using the BLASTX results against non-redundant (nr) protein database. These steps reduced the number of contigs from 240,915 to 98,317 transcripts for *Ib* and from 366,513 contigs to 275,044 transcripts for *It*. The percentage of redundant contigs was 40.8% and 75.0% for *Ib* and *It*, respectively. The filtered transcripts were used for further analysis. Likely coding sequences (ORFs) were predicted from filtered Trinity transcripts using TransDecoder located within trinity-plugins. This helped to predict longest putative ORFs from set of transcripts, out of which best scoring ORFs were selected based on Markov model (log likelihood ratio based on coding/non-coding) in each of six possible reading frames. Thus, ORFs were predicted from the filtered transcripts of both *Ib* and *It*. We also extracted FPKM values generated by RSEM for the genes of interest.

### Identification of paralogous and orthologous groups

The best coding ORFs were used for identification of orthologs using OrthoMCL [72]. All proteins derived from best coding ORFs were first compared against each other by all-vs-all BLASTP search. BLAST was performed using BLOSUM62 matrix, e-value cutoff of 1.0E-5, and masked for low complexity regions. For all matching pairs of sequences “percent match length” score was calculated and all pairs with less than 50% scores were eliminated. A network of such sequence pairs across and within species was used to determine orthologous (similar set of sequences between *Ib* and *It*) and paralogous (similar set of sequences within *Ib* or *It*) sequences. All ortholog, paralog pairs identified were then clustered using Markov Clustering (MCL) program (orthoMCL). Cluster group IDs for both paralogs and orthologs are shown in Additional files 3, 4 and 5.

### Functional annotation of transcripts

The filtered transcripts were classified into functional categories using different databases and tools. BLASTX was carried out of *Ib* and *It* transcripts against non-redundant database (nr), Arabidopsis protein database (The Arabidopsis Information Resource, TAIR10), Cassava protein database (ftp://ftp.jgi-psf.org/pub/compgen/phytozome/v9.0/Mesculenta) and potato protein database (ftp://ftp.jgi-psf.org/pub/compgen/phytozome/v9.0/Stuberosum); and BLASTN against the sweet potato gene index database (https://research.cip.cgiar.org/confluence/display/SPGI/Home). Both BLASTX and BLASTN were carried out with e-value cutoff of 10–3 and other default parameters. Transcripts with the best coding ORFs were predicted and additional annotation from trinotate was generated using databases such as Swissprot, PFAM, Protein signal prediction as well as EMBL Uniprot eggNOG and gene ontology (GO). Protein related information was added from PANTHER database based on BLAST hits to the Arabidopsis database. GO classification analysis was carried out using the GO Slim database [58] again based on Arabidopsis database. Based on level 3 category of GO classification, the transcripts were grouped into biological process, molecular function, and cellular component. The percentage of transcripts involved in each category was calculated based on the number of transcripts observed for individual functions versus the total number of transcripts (Fig. 4). Pathway information was added using information from TAIR10 database and DAVID (Fig. 5) [59, 60].

### Quantitative Reverse-transcriptase PCR (qRT-PCR)

qRT-PCR analysis was conducted using StepOnePlus (Applied Biosystems, Carlsbad, CA); cDNA was synthesized from the total RNA used for sequencing analysis. Two-step cDNA conversion was carried out using a high-capacity cDNA reverse transcription kit according to the manufacturer’s instructions (Life Technologies, Foster City, CA). First-strand cDNA was synthesized using 10x reverse transcription buffer (2.0 μl), 100 mM dNTP mix (0.8 μl), 10x RT random primers (2.0 μl), MultiScribe reverse transcriptase-50 U/μl (1.0 μl), and RNA (500 ng). The reaction was carried out at 25 °C for 10 min., followed by 37 °C for 120 min. and 85 °C for 5 min. Subsequently, second-strand cDNA synthesis was carried out in a 10-μl reaction mixture consisting of 1.0 μl 1:1 diluted first-strand cDNA, 2x Fast SYBR Green mix (5.0 μl), 900 nM of each primer (0.8 μl), and 2.4 μl of nuclease-free water using MicroAmp optical 96-well reaction plates. The experiment was conducted with three biological replicates and two technical replicates for both *Ib* and *It*. The sweetpotato *β-tubulin* gene was used as an endogenous control, and gene expression levels were determined using the ΔΔCt method [73]; ΔΔCt was calculated by comparing the ΔCt values of *Ib* and *It*.

### Simple sequence repeats discovery

SSRs were predicted from filtered transcripts of *Ib* (*Sp1*) and *It* (*Sp2*) using MISA software (Version 1.0) with default parameters (Table 5). Primers were designed for the predicted SSRs using Primer3 [74]. The forward and reverse primers were parsed using custom Perl script followed by filtering low-complexity primer sequences using SSRfinder to obtain a unique set of primers for predicted SSRs. A custom Perl script was used to extract repeat information such as SSR type, SSR size, and SSR start and end positions for the predicted SSRs with unique sets of primers and associated annotation information for *Ib* (Additional file 9) and *It* (Additional file 10).

### Discovery of single nucleotide polymorphisms and InDels

*Ib* and *It* reads were mapped against both *It* and *Ib* genes using Burrows-Wheeler Aligner (BWA) [75]. As the read coverage was very high and SNP calling programs failed to call SNPs for very high density, Picard (Version 1.107) ‘MarkDuplicates’ was used to remove the duplicate reads. About 60% of reads were marked as duplicate by Picard and were not considered for SNP calling. SNP and InDel calling was carried out with GATK (Version 3.1.1) [76] and Samtools (Version 0.1.18) [77] with all default parameters except for the following options (--baq, --mbq=20, mmq=10, stand_call_conf=50, stand_emit_conf=30 for GATK and -Q=20, -q=10 and –C=50 for Samtools). Custom perl scripts were used for further processing to denote specifically unique and common SNPs predicted by GATK and Samtools. Additional filtering of GATK predicted SNPs were filtered with GATK filters such as FisherStrand (>60), Mapping Quality (MQ <40), HaplotypeScore (>13), MQRankSum (<−12.5) and ReadPosRankSum (<−8). InDels were filtered with additional filters such as ReadPosRankSum < −20.0 and FS > 200.0. Filtered SNPs and InDels were not removed from final set, however SNPs and InDels which passed the filters were labelled as “PASS” in “FILTER” columns while SNPs and InDels that failed to pass filters have blank “FILTER” column. Annotation and ortholog/paralog information were also added to the SNPs (Additional file 11 for *Ib* and Additional file 12 for *It*) and InDels (Additional file 13 for *Ib* and Additional file 14 for *It*).

## Additional files

Additional file 1: BLAST annotation and Gene Ontology (GO) classification of *I. batatas* (*Sp1*) contigs. (XLSX 20 MB)
Additional file 2: BLAST annotation and Gene Ontology (GO) classification of *I. trifida* contigs (*Sp2*). (ZIP 42.9 MB)
Additional file 3: Paralogous genes identified in *I. batatas* (*Sp1*). (XLSX 16 KB)
Additional file 4: Paralogous genes identified in *I. trifida* (*Sp2*). (XLSX 285 KB)
Additional file 5: Orthologous genes identified between *I. batatas* (*Sp1*) and *I. trifida* (*Sp2*). (XLSX 394 KB)
Additional file 6: Primer sequences used for quantitative reverse-transcription PCR (qRT-PCR). (XLSX 12.5 KB)
Additional file 7: Functional annotation of *I. batatas* (*Sp1*) transcripts using DAVID. (XLSX 5.85 MB)
Additional file 8: Functional annotation of *I. trifida* (*Sp2*) transcripts using DAVID. (XLSX 12.4 MB)
Additional file 9: SSRs and primers predicted in *I. batatas* (*Sp1*). (XLSX 5.29 MB)
Additional file 10: SSRs and primers predicted in *I. trifida* (*Sp2*). (XLSX 7.21 KB)
Additional file 11: SNPs identified in *I. batatas* (*Sp1*). (ZIP 190 MB)
Additional file 12: SNPs identified in *I. trifida* (*Sp2*). (ZIP 89.7 MB)
Additional file 13: InDels identified in *I. batatas* (*Sp1*). (ZIP 34.3 MB)
Additional file 14: InDels identified in *I. trifida* (*Sp2*). (ZIP 22.5 MB)
Additional file 15: Gel containing RNA samples from *I. batatas* and *I. trifida*. (DOCX 1.27 MB)

## Abbreviations

4-CL: 4-coumarate-CoA ligase
ABA: Abscisic acid
*CAD*: Cinnamyl alcohol dehydrogenase
CaLB: Calcium-dependent lipid binding protein
*CoMT*: Caffeoyl-CoA O-methyltransferase
*EPSP*: 5-enolpyruvylshikimate-3 phosphate synthase
*ERF*: Ethylene responsive transcription factor
FR: Fibrous root
*Ib*: *Ipomoea batatas*
*IbMADS*1: *Ipomoea batatas* MADS-box 1
InDels: Insertions and deletions
*It*: *Ipomoea trifida*
*KEA5*: K^+^ efflux antiporter
*KNOX*1: Class I knotted1-like homeobox
*PAL*: Phenylalanine ammonia-lyase
*RGL*: Rhamnogalacturonate lyase family protein
SNP: Single nucleotide polymorphism
SR: Storage root
SSR: Simple sequence repeat
