# Supplementary figures and images for: Comparative analysis of the root transcriptomes of cultivated sweetpotato (Ipomoea batatas [L.] Lam) and its wild ancestor (Ipomoea trifida [Kunth] G. Don)
Source: BMC Plant Biol. 2017 Jan 13;17:9. doi: 10.1186/s12870-016-0950-x (PMC5234144; doi:10.1186/s12870-016-0950-x)

Additional file 15: Gel containing RNA samples from *I. batatas* (*Ib*) and *I. trifida* (*It*)


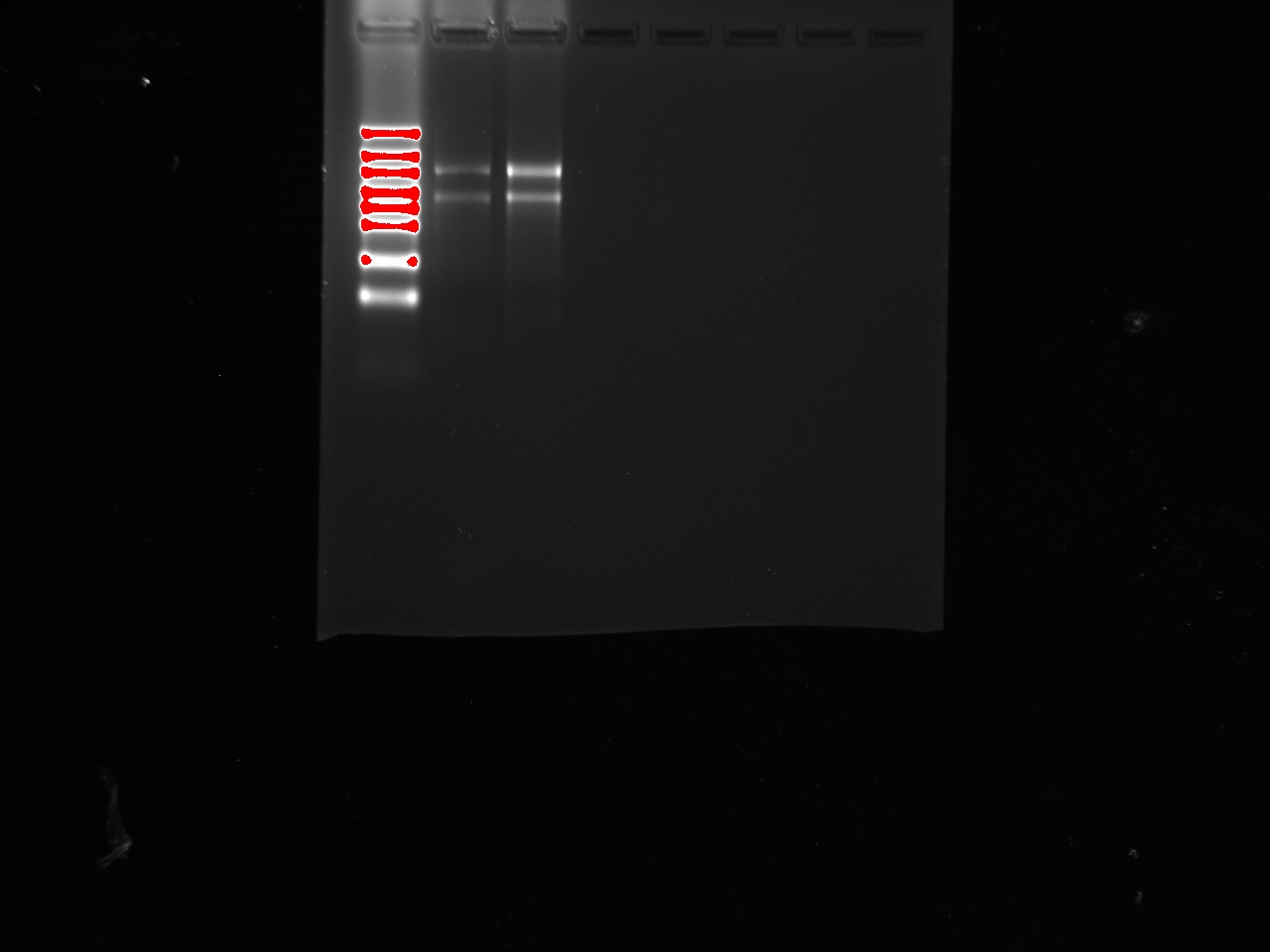


***Ib***

***It***

M

Supplement: Additional file 15: — Gel containing RNA samples from I. batatas and I. trifida. (DOCX 1.27 MB) [file 12870_2016_950_MOESM15_ESM.docx]
